# Role of Resonance Modes on Terahertz Metamaterials based Thin Film Sensors

**DOI:** 10.1038/s41598-017-07720-9

**Published:** 2017-08-04

**Authors:** Maidul Islam, S. Jagan Mohan Rao, Gagan Kumar, Bishnu P. Pal, Dibakar Roy Chowdhury

**Affiliations:** 1 Department of Physics, Indian Institute of Technology Guwahati, Guwahati, 781039 Assam India; 2 Mahindra Ecole Centrale, Jeedimetla, Hyderabad, 500043 Telengana India; 3 School of Engineering and Applied Sciences, Bennett University, Greater Noida, 201308 India

## Abstract

We investigate thin film sensing capabilities of a terahertz (THz) metamaterial, which comprises of an array of single split gap ring resonators (SRRs). The top surface of the proposed metamaterial is covered with a thin layer of analyte in order to examine various sensing parameters. The sensitivity and corresponding figure of merit (FoM) of the odd and even resonant modes are analyzed with respect to different thicknesses of the coated analyte film. The sensing parameters of different resonance modes are elaborated and explained with appropriate physical explanations. We have also employed a semi-analytical transmission line model in order to validate our numerically simulated observations. Such study should be very useful for the development of metamaterials based sensing devices, bio-sensors etc in near future.

## Introduction

Recently metamaterials have emerged as an enthusiastic research area in different portions of the electromagnetic spectrum from radio-frequencies to terahertz^1, 2^, infrared region^3^ and optical frequencies^4–6^. The metamaterials have found promising applications in diverse areas viz. medical diagnostics, food quality control, bio-sensing, detection of noxious gases, development of ultra-high speed communication devices etc^7–11^. The concept of metamaterials was first demonstrated in the microwave region, but it was soon extended to the other regions of the electromagnetic spectrum including terahertz. THz frequencies occupy a very narrow band of frequencies (0.1–5 THz) and can result in very interesting phenomenon and applications^12–16^ in term of light matter interactions. Due to longer wavelengths and therefore, it is relatively easier to fabricate metamaterials for THz as meta-molecule or split ring resonator (SRR) of typically few tens of microns. The terahertz metamaterials have widely been examined in last few years. The researchers around the world have investigated several important applications of terahertz metamaterials which include THz modulation^17, 18^, polarization rotation^7^, development of broadband photonic devices^19, 20^, resonance bandwidth enhancement^17, 21^, resonance mode hybridization^19, 20, 22–27^, electromagnetic induced transparency^28^, active chirality^23, 29–31^, sensing^32–35^ etc.

The sensing of analytes at terahertz frequencies is an intriguing area of research at the present time. Several sensing schemes have been devised by the researchers for the detection of analytes which include absorption spectroscopy, frequency shift, refractive index sensing, etc. The frequency shift sensing techniques^36, 37^ has emerged as one of the very sensitive and promising tool in sensing several analytes and their precursors. In the terahertz sensing of analytes, the design of the geometry is also very important. The sensing of analytes have been employed using both waveguide and metamaterial^38–40^. In the waveguide geometry, Theuer *et al*. examined the detection of analytes with greater sensitivity by employing cylindrical waveguide geometry^41^. Recently, You *et al*. have investigated nanofilm sensing using terahertz plasmonic waveguides by deploying an array of metal rods^42^. In that work, evanescent THz field in the metal rods is used to detect the phase variations of the surrounding analytes. Lo *et al*. have performed terahertz spectroscopic transmission measurements on porous silicon substrate, which can capture and collect analyte in the pores more effectively and sense them with greater sensitivity^43^. Terahertz metamaterials have also emerged as the potential candidate for thin film detection of analytes and several designs have been employed for greater sensitivity of the analytes. In this context. Singh *et al*. have reported terahertz sensing with high- quality factor resonances in metasurfaces^11^. The line widths of such resonances are extremely narrow and results in greater sensitivity of the analytes. In the context of bio-sensing, Xu *et al*. introduced gold nanoparticles into the terahertz metamaterials to improve the sensitivity of the protein detection^32^. The results indicate that the introduction of gold nanoparticles with high refractive indices result in the detection with enhanced sensitivity. Despite several research efforts and their outcomes, a comprehensive study of sensing capabilities of different resonance modes in terahertz metamaterials have not yet been reported in the literature to the best of our knowledge. In this work, we have explicitly focused on this aspect and have reported the usefulness of sensing performances of the odd and even resonance orders in case of a single split gap ring resonator (relatively simple geometry) based THz metamaterials.

In this paper, we examine the sensing capabilities of various resonant modes supported by the metamaterial geometry comprising of single split gap ring resonators. For our study, we have used loss-less analyte of different thicknesses over and above our metamaterials structures. The refractive index of the coated thin film is varied in order to calculate the sensitivity and figure of merit of the different resonance modes. The paper is organized as follows: first, we examine terahertz transmission properties of the designed metamaterials for the orthogonal polarizations of the incident terahertz i.e. parallel and perpendicular to the split gap. Then, we vary refractive index of the analyte film for the different thicknesses and calculated sensitivity as well as the corresponding figure of merit (FoM) to derive the sensing capabilities of different resonance modes. Next, we introduce a semi-analytical transmission line (TL)-RLC circuit model to validate and analyze numerical observations. The results are summarized in the concluding section.

## Results

### Metamaterial design and numerical simulations

**Figure 1:**
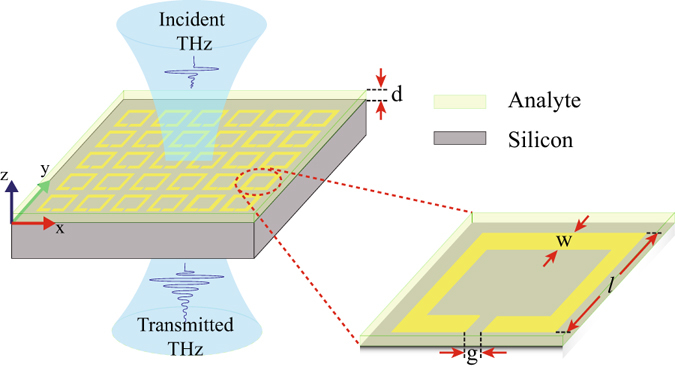
Schematic of planar Terahertz metamaterial geometry consisting of SRRs on silicon substrate. A single unit cell consists of SRR of gold metal (magnified for a closer view). The yellow region represents SRR. SRR has outer dimensions of *l* × *l* = 36 *μm* × 36 *μm*. The *w* and *g* in the schematic stand for the width and the split gap of SRR, respectively. Parameter *d* represents thickness of the analyte, which can be varied. Green coloured *y*-axis represents polarization direction of the electric field of incident Terahertz beam.

The optimum design of the terahertz metamaterial (THz) is highly crucial to sense an analyte with greater sensitivity. Our design consists of 2 dimensional array of periodically arranged single split gap ring resonator (SRR). A thin layer of analyte is coated on top of the SRR surface. We have considered analyte as coating material which is transparent to terahertz and exhibits no loss. To find out sensitivity, we vary the thickness of the analyte as *d* = 2, 4, 5, 6, …, 14, 15, 16,…, 20 *μm*. A schematic of the proposed configuration is shown in Fig. 1. We have taken silicon as the substrate and on its surface SRRs are periodically placed with a periodicity of 46 *μm* in both the *x*- and *y* - directions. The SRRs are made of gold with thickness 200 *nm* and outer dimensions for length and breadth as 36 *μm* × 36 *μm*. The capacitive gap (*g*) and line width (*w*) of the resonator are assumed to be of 4 *μm* each. The above geometrical parameters are kept constant throughout our study thereafter. In our study, we have examined terahertz transmission through the sample for both the orthogonal polarizations. In one set of simulations, electric field polarization is assumed to be parallel to the split gap of SRR and in the other case, the polarization is taken to be perpendicular to the gap. For the case of THz polarization parallel to the gap, we get two resonances, called as 1^*st*^ and 3^*rd*^ order resonances whereas in case of other polarization, a single resonance appears between the 1^*st*^ and 3^*rd*^ order resonances, which is termed as the 2^*nd*^ order resonance^44^. For our numerical study we have used commercially available numerical software, CST Microwave Studio. In our numerical simulations, tetrahedral meshing has been adopted for our configured geometry with periodic boundary conditions. The waveguide ports for the source and detector are employed. The electrical properties of the analyte and the silicon substrate in our simulations are defined with the electrical permittivities of *ε* = 3.5 and *ε* = 11.9, respectively. The terahertz transmission properties and corresponding sensing characteristics are discussed in the following section.

**Figure 2:**
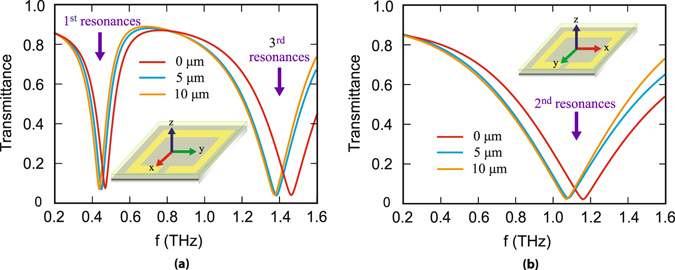
Numerically calculated THz transmittance through the metamolecule with and without the analyte as described in this work. (**a**) Shows transmittance for polarization parallel to split gap which yields two resonances 1^*st*^ and 3^*rd*^ order; (**b**) Depicts the same for polarization perpendicular to the gap, which yields 2^*nd*^ order resonance.

We examine terahertz transmittance through the designed metamaterials with and without analyte layers. A plane polarized THz radiation is incident onto the top of the metamaterial surface. The transmittance through the designed metamaterial configuration is examined for the two orthogonally polarized incident terahertz waveforms. This results in the excitation of odd and even order resonances as shown in Fig. 2. The results are shown in Fig. 2 for three different thicknesses of the analyte film. In Fig. 2, red traces represent the case for *d* = 0 *μm* i.e. when there is no analyte layer on the designed metamaterial. In this case 1^*st*^, 2^*nd*^ and 3^*rd*^ order resonance dips occur at 0.50 THz, 1.16 THz and 1.47 THz, respectively. In the figures, the blue and orange colour traces correspond to *d* = 5 *μm* and *d* = 10 *μm*, respectively. It may be noted that as the analyte thickness is increased, the resonance frequencies of the odd as well as even order resonance modes get red shifted by different magnitudes.

**Figure 3:**
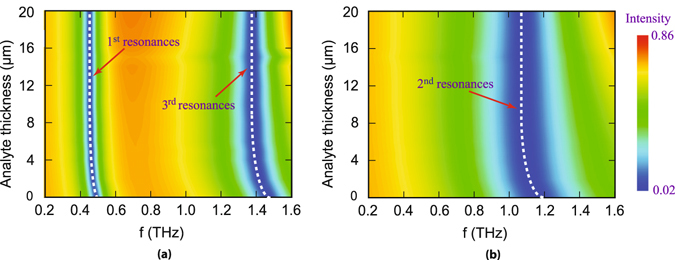
Contour plot of numerically simulated THz transmittance for different analyte thicknesses. Color bar shows the magnitude of transmission intensity. (**a**) Represents contour plot for polarization parallel to the split gap which clearly shows two blue colored regions around white dashed line for 1^*st*^ and 3^*rd*^ order resonances, respectively; (**b**) Depicts the same for polarization perpendicular to split gap i.e. for 2^*nd*^ order resonance.

In order to comprehensively understand the reason(s) behind shift in characteristic resonance dips with variation in thickness of the analytes, we numerically analyze THz transmission properties of the metamaterials with varying thickness of the analyte layer. The results are depicted in the form of the contour plots shown in Fig. 3. Figure 3(a) shows the transmittance when the polarization is parallel to the split gap for different analyte thicknesses whereas, Fig. 3(b) shows the transmittance results when the polarization is perpendicular to the gap. In these plots, we have considered the frequencies ranging from 0.2 THz to 1.6 THz to accommodate the odd and even resonance modes of the designed metamaterials covered with analyte of variable thickness from *d* = 0 *μm* to *d* = 20 *μm*. The intensity of the terahertz transmission is represented by the different colors of the contour plots. The positions of the resonance dips are indicated with the dotted white color trace inside the contour plots. It is apparent from the plots that frequency shift in the resonance dip is prominent only up to about 14 *μm* thickness of the analyte layer. It may be noted that the shift of resonance frequency is predominantly caused by the change in the capacitance of different resonance modes. We have intentionally kept the size of the resonators fixed therefore the parameter affecting resonance shift is due to capacitance alone. Capacitance of the resonance depends on the near field distribution of the electric field lines. These field lines are highly concentrated close to the resonator and recedes away from the resonator. Beyond a certain distance (14 *μm* in case of our resonator) from the resonator the electric field lines almost vanish and insignificant. Therefore presence of any analyte beyond 14 micron (in our case) from the resonator has virtually no influence/impact on the capacitance. Hence frequency shifts saturate around 14 *μm* in our study here. This is because of the limited spread of the electric field lines surrounding the resonator.

**Figure 4:**
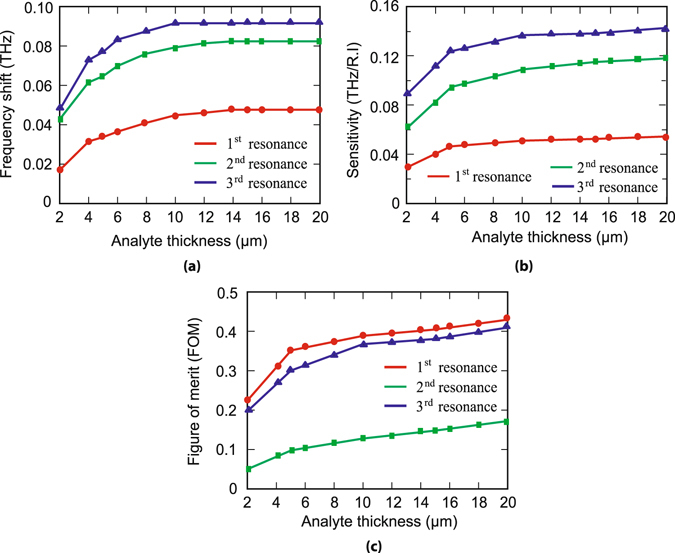
Frequency shift, sensitivity and calculated figure of merit. (**a**) Shows plot of frequency shift corresponding to different analyte thickness with respect to the resonance frequency when there is no analyte for three different resonances. (**b**) and (**c**) depicts the sensitivity and FoMs plots, respectively for different thicknesses corresponding to three different resonances.

Further, we examine more specifically, the shift in the resonance frequency of the odd and even order modes with respect to the intrinsic resonances (i.e. without any coated layer) for different thicknesses of the coated analyte layer. The results are shown in Fig. 4(a). As the analyte thickness is varied, we observe a corresponding shift in the resonance frequencies for each mode. Initially, the resonance frequency shift increases monotonically with the analyte thickness, however around *d* = 12 *μm*, it attains almost a constant value. In this figure, three different coloured traces i.e. red, green and blue colour represent frequency shift plots for 1^*st*^, 2^*nd*^ and 3^*rd*^ order resonances, respectively. Next, we calculate the sensitivities corresponding to the odd and even order resonances. The sensitivity of the metamaterial is closely related to the refractive index or the dielectric constant of the ambient material. We have derived the sensitivities for the analyte thicknesses of *d* = 2, 4, 5, 6,…, 14, 15, 16,…, 20 *μm*. In order to find out sensitivity, we varied the refractive index of analyte as *n* = 1, 2, 3 and 4 for each of the thicknesses. As we change refractive index of the film, we get a distinct resonance frequency shifts w.r.t the resonance frequency when there is no analyte i.e. *d* = 0 *μm*. We have plotted refractive index versus frequency shifts and get a linear line. The slope of this straight line indicates the sensitivity, which has the unit of THz per unit refractive index i.e. (THz/R.I.). Figure 4(b) shows the plot of the calculated sensitivities for different thicknesses corresponding to 1^*st*^, 2^*nd*^ and 3^*rd*^ order resonances.

**Figure 5:**
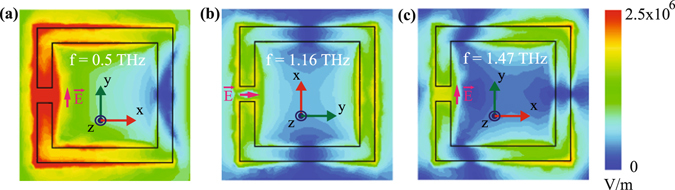
(**a**),(**b**) and (**c**) represent the Electric field profiles of 1^*st*^, 2^*nd*^ and 3^*rd*^ resonances at 0.5 THz, 1.16 THz and 1.47 THz, respectively. The green colored y-axis signifies the polarization direction of the incident electric filed.

Further, we analyse the figure of merit (FoM) of the three resonance modes w.r.t different thicknesses of the analyte film. FoM is defined as the ratio of sensitivity and full width at half maxima (FWHM). In calculating FoM, we first calculated FWHMs for different analyte thicknesses using transmittance values of the transmission output. Figure 4(c) shows the plot of FoMs for different analyte thicknesses. In this figure, the red, green and blue coloured traces represent FoM plots for 1^*st*^, 2^*nd*^ and 3^*rd*^ order resonances, respectively. One may notice that FoM increases with the increase in the analyte thickness. 1^*st*^ and 3^*rd*^ order resonances demonstrate higher FoM values compared to the 2^*nd*^ order resonances. So, electric field polarization parallel to the split gap is more useful as a sensing device than the polarization perpendicular to the gap. In our designed metamaterials, we have intentionally kept the size of the resonators constant which results in constant value of inductance. However, because of the change in coated material thickness or utilizing different refractive index materials, the effective capacitances of the split ring resonators can change. This affects the odd order resonance modes in larger extent compared to the even order modes, hence we observe higher values of FoM in case of the odd order resonances. In order to validate our physical explanation, we have monitored the electric field profiles at the 1^*st*^, 2^*nd*^ and 3^*rd*^ order resonances for the intrinsic MMs. The induced field distributions are shown in Fig. 5. The field is strongest in the split gap at 1^*st*^ order resonance (Fig. 5(a)) while at the 2^*nd*^ and 3^*rd*^ order resonant frequencies, the field is comparatively weakly confined, see Fig. 5(b) and (c). This clearly indicates that the effective capacitances for the odd order modes are more sensitive to the permittivity of the coated materials compared to the even order modes.

### Semi-analytical Transmission line model

**Figure 6:**
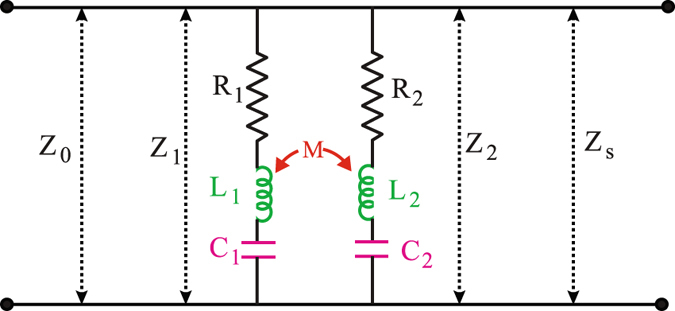
Schematic of TL-RLC circuit model. The circuit components *R*_1_, *L*_1_, *C*_1_ represent resistance, inductance and capacitance related to lower order resonance and *R*_2_, *L*_2_, *C*_2_ represent the same related to higher order resonance. *M* is the mutual inductance, responsible for coupling between resonances. *Z*_1_ and *Z*_2_ are impedances due to two circuits, respectively whereas *Z*_0_ and *Z*_*S*_ represent impedances of free space and silicon substrate, respectively.

In order to confirm the resonant behaviour of our designed metamaterials, we employ a semi-analytic transmission line (TL) - RLC model specific to our geometry^45^. RLC stands for resistance, inductance and capacitance, respectively. The circuit model of our geometry under the transmission line theory is shown in Fig. 6.

**Figure 7:**
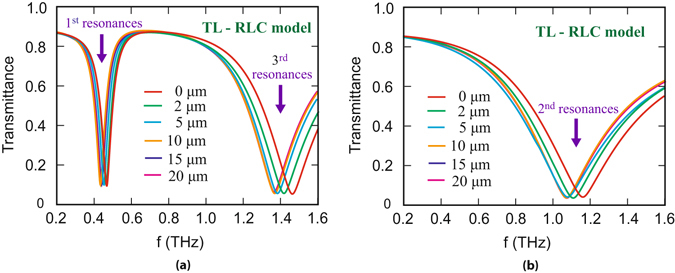
Terahertz transmittance through the typical metamaterial geometry obtained from TL-RLC circuit model for different thicknesses of analyte. The results are in good agreement with the numerical findings. (**a**) Terahertz transmittance for polarization parallel to the split gap of SRR. (**b**) Represents the transmittance for polarization perpendicular to the gap of SRR.

The results of the transmittance from the model are shown in Fig. 7(a) and (b). Figure 7(a) corresponds to 1^*st*^ and 3^*rd*^ order resonances whereas Fig. 7(b) corresponds to 2^*nd*^ order resonance. In the figure different coloured plots correspond to transmittance from the TL-RLC circuit model for different analyte thicknesses. It is noticed that they predict a similar resonant behaviour and confirm our numerically simulated results for certain values of resistance, inductance, capacitance and mutual inductance for typical SRR design. The details of the analytical transmission line model are as follows:

We assumed SRR as an equivalent RLC circuit where it is typically considered that split gap corresponds to the capacitive part, the SRR loop corresponds to the inductive part and the internal reactance of SRR is represented by the resistance part. Our numerical findings show us three resonances, 1^*st*^ and 3^*rd*^ order resonances in together for polarization parallel to split gap and 2^*nd*^ order resonance alone for polarization perpendicular to the gap. The resonant frequencies of a RLC circuit model always inversely depend on the square root of the product of inductance and capacitance. Keeping this in mind, we take two inductively coupled RLC circuits in parallel combination. In order to fit the 1^*st*^ and 3^*rd*^ order resonances together we take *L*_1_, *C*_1_ values from 1^*st*^ RLC circuit, which correspond to the 1^*st*^ resonance and *L*_2_, *C*_2_ values from the 2^*nd*^ RLC circuit, which correspond to the 3^*rd*^ resonance.

These two resonances are mutually coupled through the mutual inductance *M*. Next, to match the 2^*nd*^ order resonance with the numerical results we adjust all the circuit parameters *R*_1_, *L*_1_, *C*_1_, *R*_2_, *L*_2_, *C*_2_ with a suitable mutual inductance (*M*) value. From the circuit model shown in Fig. 6, one can calculate the circuit impedance (*Z*_*ct*_) as,

1 $${Z}_{ct}(\omega )=\frac{{Z}_{1}{Z}_{2}+{\omega }^{2}{M}^{2}}{[{Z}_{1}+{Z}_{2}-2j\omega M]},$$

where, *ω* and *M* represent angular frequency and mutual inductance respectively. *Z*_1_ and *Z*_2_ correspond to the impedances due to the 1^*st*^ and 2^*nd*^ RLC circuits, respectively. These impedances can be written as:

2 $${Z}_{1}={R}_{1}+j(\omega {L}_{1}-\frac{1}{\omega {C}_{1}}),$$

3 $${Z}_{2}={R}_{2}+j(\omega {L}_{2}-\frac{1}{\omega {C}_{2}}),$$

One can note that the circuit impedance *Z*_*ct*_ does not include the impedance due to the substrate. In Fig. 6, *Z*_0_ and *Z*_*S*_ represent impedances of free space and silicon substrate, respectively. The values of *Z*_0_ and *Z*_*S*_ are 377 ohm and 103 ohm, respectively. The overall impedance *Z*(*ω*) of our typical design including the effect of the *Z*_*ct*_ and *Z*_*S*_ in parallel combination can be written as

4 $$Z(\omega )=\frac{{Z}_{S}({Z}_{1}{Z}_{2}+{\omega }^{2}{M}^{2})}{{Z}_{S}({Z}_{1}+{Z}_{2}-2j\omega M)+({Z}_{1}{Z}_{2}+{\omega }^{2}{M}^{2})},$$

The normalized transmission amplitude, *t*(*ω*) of this transmission line-RLC circuit model is given by

5 $$t(\omega )=\frac{2Z(\omega )}{{Z}_{0}+Z(\omega )},$$

We used equation (5) to calculate the transmittance and predict resonant frequencies due to the parallel and perpendicular polarization with the split gap for certain specific values of resistance, inductance, capacitance and mutual inductance, which are defined in Tables 1 and 2. The values of *R*_1_, *L*_1_, *C*_1_, *R*_2_, *L*_2_, *C*_2_, and *M* are obtained by fitting the transmission amplitude from the simulation using equation 5. One can notice that the calculated transmittance is in good agreement with the numerical simulations.

**Table 1:** For 1^*st*^ and 3^*rd*^ resonances for different thicknesses of the analyte.

Thickness (*μm*)	*R*1 (ohm)	*L*1 (pH)	*C*1 (fF)	*R*2 (ohm)	*L*2 (pH)	*C*2 (fF)	*M* (pH)
0	10	135	0.75	8	34	0.366	−15
2	10	140	0.778	8	35	0.38	−15
5	10	141.8	0.8	8	37.1	0.37	−15
10	10	142	0.825	8	38	0.37	−15
15	10	142.2	0.83	8	38	0.37	−15
20	10	142.2	0.83	8	38	0.37	−15

**Table 2:** For 2^*nd*^ resonances for different thicknesses of the analyte.

Thickness (*μm*)	*R*1 (ohm)	*L*1 (pH)	*C*1 (fF)	*R*2 (ohm)	*L*2 (pH)	*C*2 (fF)	*M* (pH)
0	10	39.7	0.49	10	31.7	0.62	−1.5
2	10	41.9	0.5	10	33.56	0.63	−1.5
5	10	42.9	0.51	10	34.4	0.63	−1.5
10	10	43.7	0.51	10	35.7	0.63	−1.5
15	10	43.85	0.51	10	35.85	0.63	−1.5
20	10	43.85	0.51	10	35.85	0.63	−1.5

## Conclusions

In this work, we have analyzed thin film sensing potentials of the fundamental and higher order resonant modes for a simple metamaterials design consisting of single gap split ring resonators. We have calculated sensitivity and the corresponding figure of merit (FoM) for the different resonance modes supported by the designed metamaterials. We observed that the fundamental resonance mode results in the highest FoM compared to the other resonance modes. We attribute the better sensing capabilities of the fundamental resonance to its strongest electric field confinement within the split gap. Further, it is observed that the odd order resonances act as better thin film sensors compared to the even order resonances. In order to confirm our numerical findings, we have employed a semi-analytical transmission line model and found that numerical observations agree well with the theory. Because of technological ease, planar metamaterials are extremely good platform for thin film sensing including bio-sensing, temperature sensing, etc. This comprehensive study on sensing capabilities of different resonance modes of Terahertz metamaterials as described through this work should play an important role in the construction of sensing devices in future. Although we have carried out this work at Terahertz frequencies, this is a generic study and is applicable for other frequency domains of the electromagnetic spectrum too.

## Methods

We have used finite element frequency domain solver for the simulations in CST Microwave Studio package. We have employed matlab to perform the analytical modeling.

## References

[1] Pendry JB (1999). Magnetism from conductors and enhanced nonlinear phenomena. IEEE Trans. on microwav. theo. and techniq..

[2] Pendry JB (2000). Negative refraction makes a perfect lens. Phys. Rev. lett..

[3] Wiltshire MCK (2001). Microstructured magnetic materials for RF flux guides in magnetic resonance imaging. Science.

[4] Feng L (2013). Experimental demonstration of a unidirectional reflectionless parity-time metamaterial at optical frequencies. Nature mater..

[5] Xiao S (2010). Loss-free and active optical negative-index metamaterials. Nature.

[6] Vignolini, S. *et al*. A 3D optical metamaterial made by self-assembly. *Adv*. *Mat*. **24** (2012) 10.1002/adma.201103610 22021112

[7] Grady NK (2013). Terahertz metamaterials for linear polarization conversion and anomalous refraction. Science.

[8] Otsuji T (2012). Graphene-based devices in terahertz science and technology. J. of Phys. D: Appl. Phys..

[9] Knap W (2013). Nanometer size field effect transistors for terahertz detectors. Nanotech..

[10] Docherty CJ, Johnston MB (2012). Terahertz properties of graphene, *J*. *of Infra*. Mill. and THz Waves.

[11] Singh R (2014). Ultrasensitive terahertz sensing with high-Q Fano resonances in metasurfaces. Appl. Phys. Lett..

[12] Chen H-T (2006). Active terahertz metamaterial devices. Nature.

[13] Yen T-J (2004). Terahertz magnetic response from artificial materials. Science.

[14] Padilla WJ, Taylor AJ, Highstrete C, Lee M, Averitt RD (2006). Dynamical electric and magnetic metamaterial response at terahertz frequencies. Phys. Rev. lett..

[15] Chowdhury DR (2014). Excitation of dark plasmonic modes in symmetry broken terahertz metamaterials. Optics Expr..

[16] Islam M, Kumar G (2016). Terahertz surface plasmons propagation through periodically tilted pillars and control on directional properties. J. of Phys. D: Appl. Phys..

[17] Mathanker SK, Weckler, Paul R, Wang N (2013). Terahertz (THz) applications in food and agriculture: A review. Trans. ASABE.

[18] Gao W (2014). High-contrast terahertz wave modulation by gated graphene enhanced by extraordinary transmission through ring apertures. Nano lett..

[19] Zhao Q (2007). Electrically tunable negative permeability metamaterials based on nematic liquid crystals. Appl. Phys. Lett..

[20] Roy Chowdhury D, Singh R, Taylor AJ, Chen H-T, Azad AK (2013). Ultrafast manipulation of near field coupling between bright and dark modes in terahertz metamaterial. Appl. Phys. Lett..

[21] Han NR, Chen ZC, Lim CS, Ng B, Hong MH (2011). Broadband multi-layer terahertz metamaterials fabrication and characterization on flexible substrates. Optics Expr..

[22] Ekmekci E (2011). Frequency tunable terahertz metamaterials using broadside coupled split-ring resonators. Phys. Rev. B.

[23] Liu N, Kaiser S, Giessen H (2008). Magnetoinductive and electroinductive coupling in plasmonic metamaterial molecules. Adv. Mater..

[24] Prodan E, Radloff C, Halas NJ, Nordlander P (2003). A hybridization model for the plasmon response of complex nanostructures. Science.

[25] Han Z, Bozhevolnyi SI (2011). Plasmon-induced transparency with detuned ultracompact Fabry-Perot resonators in integrated plasmonic devices. Optics Expr..

[26] Chowdhury DR, O’Hara JF, Taylor AJ, Azad AK (2014). Orthogonally twisted planar concentric split ring resonators towards strong near field coupled terahertz metamaterials. Appl. Phys. Lett..

[27] Rao SJM, Kumar D, Kumar G, Chowdhury DR (2017). Modulating the Near Field Coupling through Resonator Displacement in Planar Terahertz Metamaterials. Journal of Infrared, Millimeter, and Terahertz Waves.

[28] Chiam S-Y (2009). Analogue of electromagnetically induced transparency in a terahertz metamaterial. Phys. Rev. B.

[29] Singh R, Rockstuhl C, Lederer F, and Zhang W (2009). Coupling between a dark and a bright eigenmode in a terahertz metamaterial. Phys. Rev. B.

[30] Hesmer F (2007). Coupling mechanisms for split ring resonators: Theory and experiment. phys. stat. solid. (b).

[31] Sydoruk O, Tatartschuk E, Shamonina E, Solymar L (2009). Analytical formulation for the resonant frequency of split rings. J. of appl. phys..

[32] Xu, W. *et al*. Gold nanoparticle-based terahertz metamaterial sensors: mechanisms and applications. *ACS Photonics* (2016).

[33] Xie, L., Gao, W., Shu, J., Ying, Y. & and Kono, J. Extraordinary sensitivity enhancement by metasurfaces in terahertz detection of antibiotics. *Sci*. *Rep*. **5** (2015). 10.1038/srep08671 PMC4345331 25728144

[34] Cong, L. *et al*. Experimental demonstration of ultrasensitive sensing with terahertz metamaterial absorbers: A comparison with the metasurfaces. *Appl*. *Phys*. *Lett*. **106** (2015).

[35] Shih K (2017). Microfluidic metamaterial sensor: Selective trapping and remote sensing of microparticles. J. of Appl. Phys..

[36] Chiam S-Y (2009). Increased frequency shifts in high aspect ratio terahertz split ring resonators. Appl. Phys. Lett..

[37] Chiam S-Y, Singh R, Zhang W, Bettiol AA (2010). Controlling metamaterial resonances via dielectric and aspect ratio effects. Appl. Phys. Lett..

[38] Gupta M, Srivastava YK, Manjappa M, Singh R (2017). Sensing with toroidal metamaterial. Appl. Phys. Lett..

[39] Dayal, G., Chin, X. Y., Soci, C. & Singh, R. High-Q Plasmonic Fano Resonance for Multiband Surface-Enhanced Infrared Absorption of Molecular Vibrational Sensing. *Adv*. *Opt*. *Mater*. **5** (2017).

[40] O’Hara JF (2008). Thin-film sensing with planar terahertz metamaterials: sensitivity and limitations. Optics Expr..

[41] Theuer M, Beigang R, Grischkowsky D (2010). Highly sensitive terahertz measurement of layer thickness using a two-cylinder waveguide sensor. Appl. Phys. Lett..

[42] You B (2014). Terahertz plasmonic waveguide based on metal rod arrays for nanofilm sensing. Optics Expr..

[43] Lo S-ZA, Kumar G, Murphy, Thomas E, Heilweil EJ (2013). Application of nanoporous silicon substrates for terahertz spectroscopy. Opt. Mater. Expr..

[44] Chowdhury DR (2011). Dynamically reconfigurable terahertz metamaterial through photo-doped semiconductor. Appl. Phys. Lett..

[45] O’Hara, J. F., Smirnova, E., Azad, A. K., Chen, H.-T. & Taylor, A. J. Effects of microstructure variations on macroscopic terahertz metafilm properties. *Active and Passive Elect*. *Comp*. **2007** (2007).

